# **audiomath**: A neuroscientist's sound toolkit

**DOI:** 10.1016/j.heliyon.2021.e06236

**Published:** 2021-02-10

**Authors:** N. Jeremy Hill, Scott W.J. Mooney, Glen T. Prusky

**Affiliations:** a Stratton VA Medical Center, Albany, NY, USA; b Burke Neurological Institute, White Plains, NY, USA; c Blythedale Children's Hospital, Valhalla, NY, USA; d Department of Physiology and Biophysics, Weill Cornell Medicine, New York, NY, USA

**Keywords:** Auditory stimuli, Python, Software library, Audio latency, Audio jitter

## Abstract

In neuroscientific experiments and applications, working with auditory stimuli demands software tools for generation and acquisition of raw audio, for composition and tailoring of that material into finished stimuli, for precisely timed presentation of the stimuli, and for experimental session recording. Numerous programming tools exist to approach these tasks, but their differing specializations and conventions demand extra time and effort for integration. In particular, verifying stimulus timing requires extensive engineering effort when developing new applications.

This paper has two purposes. The first is to present **audiomath** (https://pypi.org/project/audiomath), a sound software library for Python that prioritizes the needs of neuroscientists. It minimizes programming effort by providing a simple object-oriented interface that unifies functionality for audio generation, manipulation, visualization, decoding, encoding, recording, and playback. It also incorporates specialized tools for measuring and optimizing stimulus timing.

The second purpose is to relay what we have learned, during development and application of the software, about the twin challenges of delivering stimuli precisely at a certain time, and of precisely measuring the time at which stimuli were delivered. We provide a primer on these problems and the possible approaches to them. We then report audio latency measurements across a range of hardware, operating systems and settings, to illustrate the ways in which hardware and software factors interact to affect stimulus presentation performance, and the resulting pitfalls for the programmer and experimenter. In particular, we highlight the potential conflict between demands for low latency, low variability in latency (“jitter”), cooperativeness, and robustness. We report the ways in which **audiomath** can help to map this territory and provide a simplified path toward each application's particular priority.

By unifying audio-related functionality and providing specialized diagnostic tools, **audiomath** both simplifies and potentiates the development of neuroscientific applications in Python.

## 1 Introduction

The open-source software library **audiomath** makes it easy for Python programmers to synthesize, record, manipulate, edit, visualize or play sound waveforms. Since these functions are integral to a wide variety of use cases in science, engineering, art and entertainment, **audiomath**'s potential applications are many and diverse. Its development was motivated by the need for tools for designing and presenting auditory stimuli in neuroscientific research, so we will describe **audiomath** from the neuroscientist's perspective. The software is available at https://pypi.org/project/audiomath.

We created **audiomath** as part of the Burke-Blythedale Pediatric Neuroscience Research Collaboration, to support field applications of neurotechnology—specifically, EEG-based cognitive assessments in children with brain injuries, performed at the bedside. This is one component of our broader vision of a “scalable neurological assessment platform” (SNAP) which consists of multiple reusable software modules. Another published component, **Shady**, allows rendering and real-time manipulation of research-quality visual stimuli even on sub-optimal hardware [1]. Providing full documentation and support to external users is part of our strategy to ensure that modules such as **Shady** and **audiomath** remain usable and future-proof as the platform evolves. Python was chosen as the basis for this platform due to the maturity and modularity of the language, the power and minimalism of its syntax, the comprehensiveness of both its standard library and the range of high-quality packages available for free from third parties, and its consequent high prevalence in scientific communities.

We identified the need for software tools that facilitate—and, to the greatest extent possible, enable us to automate—the four main tasks enumerated below with minimal effort on the part of the programmer. In outlining these tasks, we have underlined the recurring lower-level functions that these tasks require:

1. Gathering raw material to make auditory stimuli—depending on the experiment, this might include decoding content from existing audio files, generating waveforms from simple mathematical specifications, recording sounds from a microphone, or a combination of these.
2. Composing stimuli by manipulating the sound data in memory—for example, extracting segments of interest, trimming or padding to the required length, aligning, windowing, rescaling, resampling, cleaning up, multiplexing and splicing. As part of this process visualization and playback are valuable tools for previewing the stimulus under construction. Typically, the end-point of this pipeline entails encoding the result in some recognized audio file format and saving it as a file.
3. Presenting stimuli to the subject. For this, we needed our playback functionality to meet certain criteria:
  • ease of controlling multiple overlapping stimuli independently;
  • the capability of playing multi-channel sounds (more than 2 channels);
  • programmatic control of the operating-system's overall volume (because we found that standardizing the system volume by hand, as part of an experimenter's standard operating procedure, is overly prone to omission due to human error);
  • in some applications, we need to achieve low latency, and/or minimal *variability* in latency (“jitter”)—see Section 3;
  • in other applications, we might be willing to sacrifice latency but require playback to be robust (able to tolerate the use of processing resources by other threads or processes without the sound stuttering, skipping or slowing down) and/or cooperative (i.e. to allow other processes to play sound at the same time).
4. Capturing sound during the experimental session: some experimental designs require continuous recording of sound data and encoding it to file, for example to capture verbal responses by the subject, notes dictated by the experimenter, and other events that may be relevant to subsequent data analysis.

In summary, the lower-level functions (underlined above) are: manipulation, generation, visualization, decoding, encoding, recording, and playback. In Section 2, we explain how **audiomath** enables each of these functions. In Section 3 we examine the issue of performance, defining criteria by which audio presentation performance can be judged, the factors that affect it, and outlining various approaches to the problem of recording stimulus timing. In Section 4 we describe the methods, included in the **audiomath** codebase, that we used to gather illustrative performance data from a range of hardware and operating systems, which we then report in Section 5 before concluding.

## 2 Design

The goal of any programmer's toolbox is to provide functionality at a certain level while saving the programmer from having to worry about implementation details at lower levels. This can be achieved both by writing original code and by wrapping third-party code (the purpose of wrapping being to ensure that code from different sources operates smoothly together, while exposing a consistent level of detail to the programmer who uses it). In **audiomath**, we aim at the level of detail exemplified in Listing 1. It allows the Python implementation of an idea at this level to be roughly as concise as the English-language expression of the same idea, while still being highly readable and maintainable by programmers.

**Listing 1:**
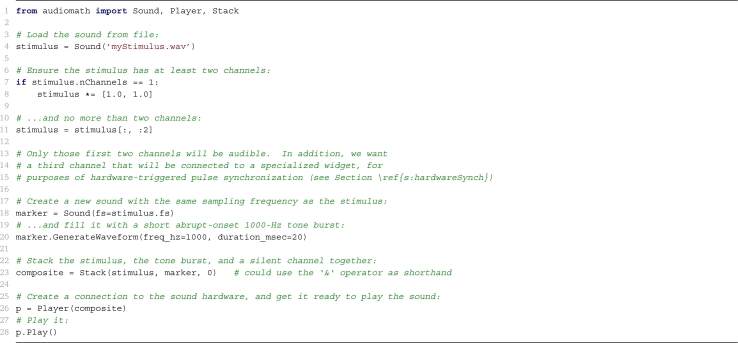
Example Python code, using **audiomath** to accomplish the goal, *“Create and present a four-channel stimulus that has the content of the file***myStimulus.wav***in the first two channels (left and right), simultaneous with a tone burst for synchronization in a third channel, and silence in a fourth.”*. This goal is expressed in 230 characters or 39 words of English. Disregarding the comments and an initial **import** statement, the listing uses 270 characters or 34 words/numbers.

The following subsections describe the way in which **audiomath** uses original code and unites the functionality of various third-party toolboxes, to implement each of its essential functions. Finally, Section 2.7 summarizes the third-party dependencies entailed in these solutions, and how **audiomath** manages them.

### 2.1 Manipulation

At its core, **audiomath** represents sounds as numeric arrays, via the (required) third-party Python package **numpy**. **numpy** is well-established, well-supported, and ubiquitous in scientific Python applications [2], [3], [4]. In **audiomath**, sound arrays are embedded in an object-oriented interface, bundling together the raw data and the associated meta-information into **Sound** objects. The name **audiomath** is inspired primarily by the ability to perform arithmetic operations on these objects using elementary syntax—for example, x = 2 * y + z makes a linear superposition of sounds. Similarly minimal and intuitive notation can be used for slicing and splicing along the time dimension, selection and multiplexing of channels, resampling, mixing, trimming, padding, rescaling, and amplitude modulation.

For more sophisticated types of manipulation, **audiomath** also provides wrappers that interface with some optional third-party audio processing utilities. For example, it can:

• Time-stretch or pitch-shift a sound (i.e. make it last longer while holding pitch constant, or change its pitch while holding duration constant). To do this, **audiomath** simply provides a convenience wrapper around the phase-vocoder implementation provided by the (optional) third-party Python package **librosa** [5].
• Process a sound via the SoX utility. As part of **audiomath**, we provide a convenience wrapper around **sox**, a third-party cross-platform command-line tool that can perform a range of different processing and editing functions on sounds. One of these, of particular interest to neuroscientists, is the “loudness” effect, which can standardize the expected perceived intensity of a sound according to international standard ISO 226 [6].

Furthermore, the easy accessibility of the raw **numpy** array inside each **Sound** object, and the ubiquity of **numpy**, makes it relatively easy for users to implement any sound data transformation or analysis method that is not already implemented in **audiomath**, either from scratch or using third-party audio processing packages such as **librosa** and **scipy.signal**.

### 2.2 Generation

In its **Signal** sub-module, **audiomath** provides routines for synthesizing simple stimulus waveforms such as clicks, sine-waves, square-waves, sawtooth-waves and triangle-waves, with or without antialiasing, and for modulating their amplitude with other waveforms, including windowing/tapering functions. There is also support for playback of sounds that are functionally generated on-the-fly (i.e. while playing).

### 2.3 Visualization

Sound waveforms, and/or their amplitude or power spectra, can be plotted provided the (optional) third-party Python package **matplotlib** is installed. Like **numpy**, **matplotlib** is an almost-ubiquitous standard, used throughout the world by scientists of all fields [4], [7], [8].

### 2.4 Decoding and encoding

Using routines that are already part of Python's standard library, **audiomath** can read uncompressed **.wav** files into memory, and write them back out to file. It can also decode a wide variety of other audio formats, or extract the sound track from a variety of video formats, using the third-party C library **AVbin** [9]—**AVbin** binaries are included in the package, for the most common platforms. Non-**.wav** formats can also be encoded and written to file—under the hood, this uses **audiomath**'s own wrapper around the popular third-party command-line utility **ffmpeg**, which must be installed separately. (Alternatively, **sox** may be used—again, this must be installed separately.)

### 2.5 Recording

To access the audio input and output hardware, **audiomath** ships with a back-end implementation based on the third-party cross-platform C library **PortAudio** [10], binaries for which are included for a range of common platforms. This allows sound to be recorded either into memory or direct to file. Recording direct to file additionally requires a separate installation of the **ffmpeg** or **sox** command-line utilities. The **PortAudio** library also enables flexible and precise playback (see next section).

### 2.6 Playback

The default back-end for playback is the **PortAudio** library (also used for recording—see previous section). Across a range of devices, we have found that this provides flexibility and reasonable performance (see the results of Section 5). An alternative back-end, based on the optional third-party package **psychtoolbox**, is also included—this enables access to **PsychPortAudio**, a customized version of the **PortAudio** library tailored for precise timing [11], [12]. Users can switch to the **PsychPortAudio** implementation if they want to reduce jitter even further.

Programmers may also use **audiomath** to control the operating-system's overall volume. This is accomplished via bindings to Applescript on macOS, to PulseAudio command-line utilities on Linux, or to Microsoft's Component Object Model on Windows (which requires the third-party Python packages **comtypes** and **psutil**).

### 2.7 Summary of **audiomath**'s third-party dependencies

To implement each of the required functions described in the foregoing sections, **audiomath** brings together functionality already implemented by various third-party developers. Some of these dependencies are included in the package when it is installed, some others are installed automatically when the user installs **audiomath** via a package manager such as the ubiquitous **pip**, and some others are left to the user's discretion. For reference, they are summarized in Table 1.

**Table 1:** This table lists the third-party dependencies of **audiomath**. The recommended way of installing **audiomath** is to use the command **python -m pip install audiomath** and this will handle the dependencies listed as “automatically installed” and “included”. Optional dependencies can be installed at the user's discretion, using **pip** for Python packages (for example with **python -m pip install psychtoolbox**) and separate methods (independent of Python) for the command-line utilities.

Name	Type	Role
**Automatically installed alongside audiomath on all platforms:**		
**numpy**	Python package	required for everything **audiomath** does
**Automatically installed alongside audiomath on Windows:**		
**psutil**	Python package	required for controlling the system volume on Windows
**comtypes**	Python package	required for controlling the system volume on Windows
**Included in the audiomath package, ready-compiled for common platforms:**		
**PortAudio**	binary (C library)	enables recording and playback
**AVBin**	binary (C library)	enables sounds to be decoded from common compressed audio and video file formats
**Optional add-ons that can enhance audiomath's functionality:**		
**matplotlib**	Python package	enables plotting of waveforms and spectra
**psychtoolbox**	Python package	enables playback with very low jitter
**librosa**	Python package	enables pitch-shifting and time-stretching; (as well as providing a library of other sophisticated analysis and processing functions)
**ffmpeg**	command-line utility	enables **audiomath** sounds to be written to file in a wide range of encoded formats
**sox**	command-line utility	allows various effects to be applied to sound; enables **audiomath** sounds to be written to file in some encoded formats

With the exception of **numpy**, **audiomath**'s functionality degrades gracefully in the absence of these dependencies. For example, if the user's operating system is not among the platforms already supported by the included **AVBin** binaries (currently: 64- and 32-bit Windows, 64-bit macOS and Linux), then **audiomath** will only be able to read audio data from uncompressed **.wav** files, but other functions will still work; or, if the user chooses not to install **matplotlib**, plotting will be disabled but no other functions will be compromised.

## 3 Understanding playback performance: a primer for neuroscientists

When a computer program issues the command to play a sound (or modulate it, or stop it), the command may return and allow the program to proceed within microseconds; however, the physical sound will not start (or change, or stop) until much later. This delay, referred to as audio latency, reflects the time taken for the operating system's sound libraries, drivers and hardware to process the command. Latencies are highly variable between hardware/driver/operating-system configurations—from a few milliseconds to a few hundred milliseconds. They are also somewhat variable from one repetition to another on the same setup—we define “jitter” as the within-setup standard deviation of latencies measured under uniform conditions.

Latency and jitter both matter, for different reasons, to neuroscientists. Additional performance criteria may also interact with one's latency settings. We outline multiple performance criteria and their significance in Section 3.1 below. Of these criteria, uncertainty in stimulus timing, in the form of jitter, tends to demand the largest amount of energy when one is engineering neuroscientific experiments and applications. In Section 3.2 we outline different methods for recording the timing of auditory stimuli for the purpose of reducing uncertainty. In Section 4 we describe how **audiomath** unifies these methods to provide a measurement tool for latency and jitter. In Section 5 we report results of such measurements, illustrating some of the factors that affect performance.

### 3.1 Performance criteria and their significance in neuroscience

In the following subsections we define multiple performance criteria—latency, jitter, robustness, cooperativeness, multi-channel capability and programmability—and briefly highlight the significance of each. These criteria may matter to differing extents, and they may be more or less easy to achieve, depending on whether the application is deployed in a laboratory or “in the field” (for example, as a bedside test in a clinical setting). In field applications there may be less scope for dedicating specialized hardware to each separate task, higher likelihood of having to use sub-optimal hardware (often chosen for portability rather than performance), and greater imperative to multi-task (for example, in our pediatric applications we often present critical stimuli opportunistically, interleaved with entertainment that usually requires a browser-based video player).

**Figure 1:**
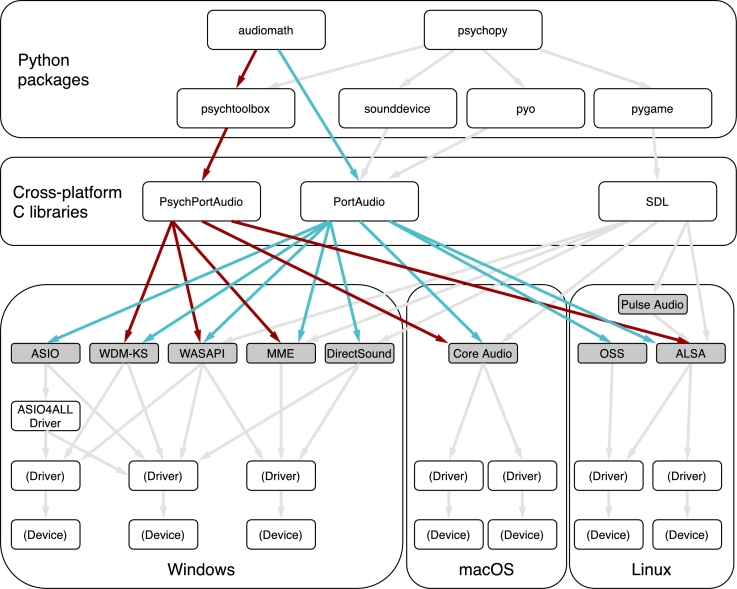
This figure shows software components that provide Python programmers with access to sound input and output hardware. We show the components that are relevant to **audiomath** and, for comparison, the audio components related to **psychopy**, which is a separate high-level package also aimed at neuroscientists. Both of them make use (directly or indirectly) of cross-platform libraries such as **PortAudio** which, in turn, wrap the various platform-specific “host APIs” (darker boxes). Each host API has advantages and disadvantages relative to the others, including the fact that the driver for a given device may support features of different host APIs to differing extents. Therefore, **PortAudio** (and the Python packages that use it, such as **audiomath**) provide users with commands by which the desired host API can be chosen and configured. The choices available to the **audiomath** user have been highlighted: blue (mid-intensity) arrows indicate options that use the standard **PortAudio** library, and red (darkest) arrows indicate options that take advantage of **PsychPortAudio**, a performance-optimized version of **PortAudio** that comes from the Psychophysics Toolbox project. Gray (lightest) arrows show connections that are either unrelated to **audiomath** or outside of the **audiomath** user's control. The figure is incomplete, in the sense that there are many other sound libraries available, unrelated to either **audiomath** or **psychopy**, and even additional host APIs that **PortAudio** does not support.

Often it is necessary to accept a trade-off between criteria. Most obviously, low latency and robustness pull in opposite directions. On the Windows platform there are further complications, as the lowest latencies are typically only available at the expense of cooperativeness. This depends on what the **PortAudio** library refers to as the host application programmer's interface or “host API”. Host APIs are platform-specific libraries that allow programmers to interface with sound drivers without writing driver-specific code.^1^ **PortAudio**, as a cross-platform library, offers an additional level of abstraction so that the programmer does not need to write host-API-specific code. It does this by rolling the selection of host API into the user's selection of sound device: for example, on a given Windows computer, device #4 might be a pair of headphones as addressed via the “DirectSound” host API, whereas device #6 might be the same pair of headphones as addressed through the “WASAPI” host API.^2^ This selection affects performance in multiple ways, especially under Windows, as we will discuss below and illustrate in Section 5. Therefore, it is valuable to be aware of the available choices, which are illustrated in Fig. 1. One motivation in making **PortAudio** the default back-end for **audiomath** was that it provides a simple way for the user to cut through the complexity of Fig. 1 and choose a host API with minimal fuss.

#### 3.1.1 Low latency

Some applications require absolute latency to be as low as possible. In neuroscience these are typically closed-loop interactive applications that are intended to mimic real-world interaction. Such applications need low latency for the same reasons musicians need it: to ensure sound perception is sufficiently tightly bound to other sensory inputs and to efference copy information from the motor system. This concern was highlighted in a study by Scarpaci et al. [14], who manipulated the latency of real-time updates to the virtual head-centric position of a sound stimulus, which changed as a function of subjects' head movements while tracking the stimulus. They measured subjects' errors in tracking, reporting equal (baseline) error rates at latencies of 4 and 18 ms, with increasing errors as latency increased further (32 ms or more). Some hardware and software configurations are capable of producing latencies below 20–30 ms and some are not. Therefore it is clear that neuroscientists would need to select and configure their computers carefully and validate their performance for this application—and indeed for applications involving *other* response modalities that might have even narrower latency tolerances.

Another advantage of lower latency is that it tends to entail lower *uncertainty* in stimulus onset time (although there are exceptions, such as the one noted in the next subsection). In other words, as absolute expected latency decreases, absolute jitter usually also decreases.

#### 3.1.2 Low jitter

Post-hoc analyses of the brain's responses to sound are anchored first and foremost in knowing *when* the sounds occurred, relative to the response data that are being collected (brain signal data, eye movements, button-presses, etc). This is where jitter matters: even if the absolute latency were high, zero jitter would mean that expected latency could be measured once and then compensated-for precisely in every analysis thereafter; conversely, non-zero jitter entails uncertainty in the timing of each individual stimulus, degrading the power of the analysis. Various approaches for recording stimulus timing, and the way in which they deal with jitter, are described in Section 3.2. Usually, jitter decreases as the absolute expected latency decreases, but there is one notable exception: when using **PsychPortAudio**, a performance-optimized version of **PortAudio** that comes from the Psychophysics Toolbox project, it is possible to *pre-schedule* a sound. Relative to the time the command is issued, the scheduled time should be completely outside the usual distribution of latencies (so, the effective latency is longer than usual) but the resulting jitter is very low, regardless of absolute latency.

#### 3.1.3 Robustness

We use the term “robust” to describe a system that plays and records clean audio without skipping, stuttering, crackling or slowing, despite concurrent use of CPU or memory resources by one's own program,^3^ or by other processes on the computer. In traditional laboratory settings, an experimenter may be able to dedicate and configure a computer (or even a more-expensive specialized audio processor) exclusively for the task of auditory stimulus presentation. In this case, the experimenter typically does not have to worry about robustness thereafter. In field applications, by contrast, robustness may be of greater concern. The lower the latency, the higher the risk of disruption—therefore, in some situations it may be necessary to run at higher latency for the sake of robustness.

#### 3.1.4 Cooperativeness

We call a process “cooperative” if, while using a given sound device, it allows other processes to use the same sound device concurrently. On Windows, the programmer must unfortunately choose: the lowest-latency host APIs (WASAPI with aggressively-configured latency settings, or ASIO,^4^ or WDM/KS^5^) are non-cooperative, whereas selecting a host API capable of cooperation (WASAPI with cooperative settings, or MME,^6^ or DirectSound) results in considerably higher latencies. In many neuroscience experiments it may actually be desirable for the main process to use the sound hardware uncooperatively, since this will prevent incidental interruptions by sounds from other processes. However, it is undesirable in more-integrated applications—consider, for example, a brain-computer interface designed purely as an access method through which users might instruct their computers to perform any normal task, including playing music or making calls.

#### 3.1.5 Multi-channel capability

Nearly all sound software, and nearly all sound output hardware, is capable of delivering two precisely-synchronized channels simultaneously: left and right. We use the term “multi-channel” to refer to anything that delivers *more than two* channels, such as a surround-sound system. Even when we need only two channels for stimulus presentation, it is sometimes critical to have more available—for example, when implementing some types of hardware-triggered pulse synchronization, as described in Section 3.2.3. In addition to demanding suitably specialized hardware, this requirement may constrain one's choice of software (before adding **PortAudio** support to **audiomath**, we tried a number of other audio toolboxes for Python, and found that many of them were not multi-channel capable) as well as one's choice of host API (on Windows, the “MME” host API is not multi-channel capable). Luckily, the market for surround-sound entertainment applications has ensured that suitably specialized portable hardware is available and well-supported—we have had good results from 8-channel dedicated USB sound adapters that cost around 30 US Dollars, in both performance tests and in our auditory brain-computer-interfacing studies [16].

#### 3.1.6 Programmability

In designing **audiomath**, we aimed to allow programmers to automate as much functionality as possible, reducing potential sources of error by cutting out manual intervention by the experimenter. However, some software configurations can undermine this. For example, Fig. 1 shows that, under Windows, sound hardware can be addressed via the ASIO host API and the third-party **ASIO4ALL** driver. This offers flexibility in configuring buffering and latency settings and can be used to achieve very low latencies. However, some of its parameters must be accessed by the end-user manually in a graphical user interface (GUI): **PortAudio** can programmatically increase buffer size relative to the GUI-configured setting, but cannot decrease it, and has no access to the influential “buffer offset” setting. Note that **ASIO4ALL** is itself built on top of the WDM/KS host API—accordingly, we have found that comparably low latencies are achievable by having **PortAudio** address WDM/KS directly, so we have eventually concluded that there is no need to use **ASIO4ALL**.

#### 3.1.7 Hardware-invariance in latency

Software intended for distribution to collaborators or customers may end up running on a wide variety of hardware. It may be desirable for performance to vary as little as possible across different hardware setups. We find that the property of invariance is itself affected by one's configuration—particularly, once again, the choice of host API on Windows (DirectSound latencies being highly variable across the setups we have measured, and WDM/KS latencies being the least variable).

### 3.2 Synchronization methods

In the following subsections, we describe four approaches to recording the timing of auditory stimuli: software logging, software-triggered pulse generation, hardware-triggered pulse generation and hardware-synchronized envelope extraction. The **audiomath** source code repository contains microcontroller code that can be used to implement software-triggered or hardware-triggered pulse synchronization, with the option to use both methods simultaneously for measuring audio latencies (see Section 4).

#### 3.2.1 Software logging

In the software logging approach, the computer merely saves a record of the times at which sound commands were issued. It requires a common clock that can be read by the stimulus presentation program and also by whatever system provides timestamps for the response data. This approach is used by most applications in the popular brain-computer interfacing platform BCI2000 [17], [18]. It does not account for audio latency or jitter: when these are critical, the best one can do is to perform a separate validation using hardware triggering to verify that jitter is low enough.^7^ Validation methods might include, for example, the method developed and reported by Wilson et al. [19] for BCI2000, or **audiomath**'s own method described below in Section 4. The assumption is that the latency and jitter are the same during validation as they will be during subsequent use without the hardware trigger. This assumption leaves us vulnerable to non-stationarity of the system's audio latency—either transient variation due to contention over processing resources with some background system task, or lasting changes due to updates in the drivers or other operating-system components (the latter means that the validation procedure must be repeated after any system update or reconfiguration).

#### 3.2.2 Software-triggered pulse synchronization

When there is no common clock that both the stimulus presentation program and the data acquisition system can read, software logging cannot be used. Instead, it may be possible to use software-triggered pulse synchronization. This relies on the fact that many biosignal recording devices can record auxiliary information time-locked to the primary signal. One or more auxiliary channels can be used to record stimulus timing—typically by marking stimulus onset with the rising edge of a 5-volt pulse. Immediately before playing a sound, the computer sends a message over a serial, parallel or network interface to a specialized synchronization device (“widget”) that generates such a pulse as soon as it receives the message. The widget is typically a simple processor that performs little or no multi-tasking, so its timing is very precise relative to that of the computer. However, the computer's half of the communication link will inevitably add a small extra amount of latency and jitter to the whole system. Other than this, the software-triggered pulse synchronization approach is no different from the software logging method, and suffers all the same problems.

#### 3.2.3 Hardware-triggered pulse synchronization

Like the software-triggered approach, hardware-triggered pulse synchronization also uses a pulse-generating widget. Instead of triggering the pulse via digital communication, the computer's audio output cable is split so that identical signals run both to the subject's headphones or speakers, and also to an analog input on the widget. The widget monitors the sound signal and generates a pulse as soon as it detects that the amplitude exceeds some threshold. This is the most accurate and precise way to localize stimulus onsets in time, provided the sound amplitude exceeds the threshold at the right moment. A potential complication arises when working with natural sounds, which may have gradual onsets to differing extents: the relative onset time of two natural sounds may then appear to change according to the (arbitrarily-chosen) threshold parameter value.^8^ This problem can be circumvented if the sound software and hardware are capable of delivering more channels than are needed for actual speakers and headphones—each stimulus can incorporate an artificial, abrupt-onset sound in the extra audio channels, whose corresponding cables are connected directly to the widget and not to the speakers.^9^ If the stimuli are monophonic, one can use inexpensive audio cable adapters to make this work even with simple stereo hardware (one channel for sound, one for synchronization). For stimuli that require two or more audible channels, one must take steps to ensure that one's hardware, drivers and host API all have multi-channel capability.

#### 3.2.4 Hardware-synchronized envelope extraction

**Figure 2:**
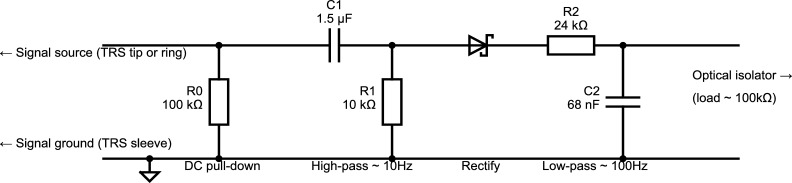
This circuit extracts the envelope of an audio signal by high-pass-filtering, then rectifying, then smoothing, so that it is suitable for recording in an auxiliary channel of a recording device with a low (200 Hz or more) sampling frequency. The circuit was designed to capture sound energy anywhere above 10 Hz, and to capture amplitude fluctuations up to 100 Hz. The resistor value R2 was chosen in the context of a 100 kΩ load on the output (from an optical isolation unit used to isolate the circuit from the EEG recording device, for safety reasons). Smaller loads (larger load resistances) lead to longer decay times of the circuit's impulse response—to bring this back into the low-double-digit millisecond range, R2 might have to be increased (and C2 reduced accordingly so that the product R2⋅C2 remains constant).

Another approach is to abandon the use of digital pulses and instead record sound intensity directly in an *analog* auxiliary channel of the data recording system, if available. (Note that if this involves plugging the audio signal into an input normally intended for sensors that contact the subject, then an optical isolator is required between the audio cable and the auxiliary input, for safety.) Usually the sampling rate of the data recorder is too low to capture the full audible frequency spectrum—if so, one can instead opt to extract and record the *envelope* of the sound, using a digital audio processing widget or even a passive circuit such as the one shown in Fig. 2. Either way, this approach allows subsequent analysis to use the envelope information in its entirety—or, if it becomes necessary to define onset as a single instant, it can be decided post-hoc how that instant is determined. We used this approach in an EEG brain-computer interfacing study [20], employing the custom-made passive circuit shown in Fig. 2 to extract the sound signal's envelope.

## 4 Materials and methods

We will report audio latency results measured using a widget that performs both software- and hardware-triggered pulse synchronization, in combination with a way of measuring the time between the two triggers. We used a PJRC Teensy [21] version 3.1 microcontroller as the basis for the widget, and added an audio jack. We loaded it with the **PlayerLatency-TeensySketch** microcontroller program provided in **audiomath**'s code repository, and used it in conjunction with the accompanying Python script **PlayerLatency.py**.

**Figure 3:**
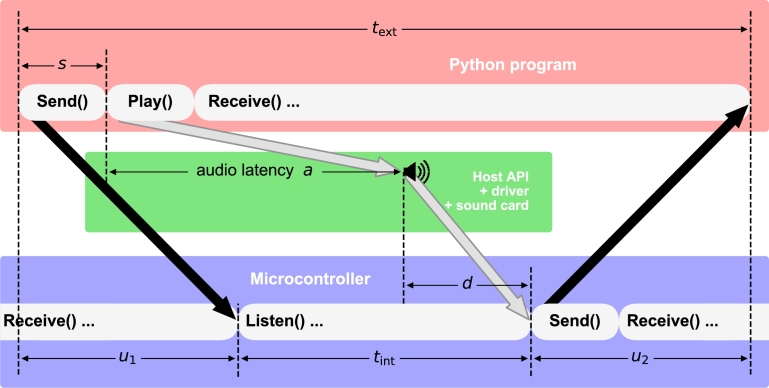
This schematic shows our system for estimating the audio latency *a* of stimulus presentation (or the overall latency *s* + *a* of a software-triggered stimulus logging and presentation system) by implementing both software- and hardware-timed triggering in a microcontroller-based timing widget. Time is on the horizontal axis. Black arrows indicate serial-port communication. The analog output of the computer's sound-card is connected to an analog input of the microcontroller. Gray arrows indicate the audio pathway: processing of the **Play()** command by the host API, driver and hardware, followed by the microcontroller's detection of the physical sound signal (which takes time *d*). Time durations *t*_ext_ and *t*_int_ are measured by the computer and the microcontroller, respectively. *u*_1_ and *u*_2_ are unknown (not directly measurable) latencies of serial-port communication. See text for further details.

Fig. 3 shows the system schematically. Immediately before issuing the command to play a sound, the computer sends a message over a serial connection to the microcontroller (first black arrow). When it receives this message, the microcontroller reads its internal clock and then begins monitoring its audio input, which is connected to the computer's analog sound output. As soon as it has detected the onset of the physical sound signal, the microcontroller reads the clock again, and sends its estimate of the elapsed time $t_{\text{int}}$ back to the computer over the serial port (second black arrow). The computer, meanwhile, measures the time $t_{\text{ext}}$ taken for the whole round trip, including the two unknown (not directly measurable) serial communication latencies $u_{1}$ and $u_{2}$. Starting from the sound's actual physical onset, it takes some small time *d* to gather evidence for detecting that the sound is present. The size of *d* can be estimated from separate measurements of $t_{\text{int}}$, taken while the sound is *already* playing on an indefinite seamless loop (in which context **Play()** commands have no additional effect on the audio output). We have $t_{\text{ext}}=u_{1}+t_{\text{int}}+u_{2}$ and $s+a+d=u_{1}+t_{\text{int}}$, where *s* is the time that it takes for the initial **Send()** command to return (depending on the computer, we found *s* to be between 0.1 to 0.4 ms, with jitter typically around one third of its magnitude). If we make the assumption that u1=u2, then we can estimate $a=(t_{\text{int}}+t_{\text{ext}})/2-s-d$. In our implementation we found *d* to be less than $\frac{1}{10}$ of a millisecond (mean 0.02 ms, SD 0.04 ms) and so we disregard it in our estimates.

## 5 Results

Performance may be affected by many factors—for example:

• the quality of one's chosen hardware;
• the way in which the sound hardware interfaces with the computer (it may be “on-board”, i.e. integrated into the computer's motherboard, or it may be a dedicated device attached either via the computer's PCI bus or USB bus);
• the quality of its driver, and which features of which host APIs the driver supports;
• the programmer's choice of host API through which to address the driver;
• the programmer's choice of latency settings (PortAudio offers the opportunity to configure a “suggested latency” setting and a buffer size, both of which will affect latency and robustness);
• the amount of resources demanded both by other threads of the sound-generating process, and by other processes.

The results of our latency measurements (using the method described in Section 4) illustrate how some of these factors affect performance. Table 2 shows the profound effect of one's choice of host API when running on Windows. The measurements were taken using one of our preferred platforms for field applications, an all-in-one computer whose sound hardware is not particularly well optimized for performance. The results are heavily influenced by one's chosen “suggested latency” setting, which is an input parameter exposed by both **PortAudio** and **PsychPortAudio**, and accordingly by **audiomath**, during the initialization of the software connection to the sound driver. (Note that the “suggestion” is not followed faithfully on Windows—the true latency is significantly higher.) It was clearly possible to set this setting *too* low, producing audible artifacts (rows 2A and 2J). Nonetheless, it was possible to achieve very low latency (<10 ms) and jitter (<1 ms) without artifacts, either using our default **PortAudio** back-end (row 2B) or using **PsychPortAudio** (row 2D). Furthermore, by increasing latency slightly, it was possible to use **PsychPortAudio**'s pre-scheduling functionality to reduce jitter even further (±0.3 ms, row 2E).

**Table 2:**
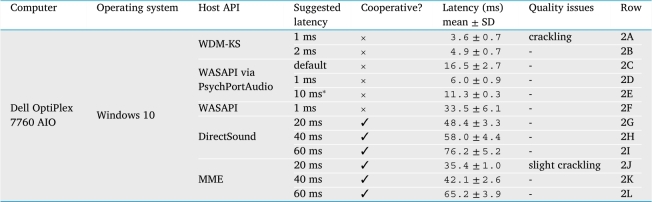
This table shows observed audio latency and jitter (mean and standard deviation of latency across 100 repetitions) measured via the headphone socket of one particular all-in-one Windows computer using the method described in Section 4. Performance varies greatly depending on the chosen host API and latency settings. “Suggested latency” refers to a configurable parameter exposed by the **PortAudio** library and by **audiomath** when initializing sound hardware, except in the case marked ^⁎^ (row 2E): this measurement was taken by “suggesting” a latency of 1 ms as in the previous condition, but then using **PsychPortAudio**'s unique pre-scheduling functionality to configure a longer latency of 10 ms, resulting in reduced jitter.

These results, and others like them from other computers, have led us to offer two sets of default settings in **audiomath**. If a program starts by announcing itself as a low-latency application by calling the **audiomath** function **LowLatencyMode(True)**, a suggested latency of 4 ms will be used by default, and on Windows the WDM/KS host API will be preferred. Without this declaration, **audiomath** defaults to more robust, cooperative settings: on non-Windows platforms, suggested latency is at least 10 ms (typically leading to actual measured latencies of around 20 ms), and on Windows, DirectSound is preferred, with a suggested latency of 60 ms (we found this setting to be more robust than lower values in the context of moderate to high concurrent CPU use).^10^ From here, therefore, we narrow our focus to consider just WDM/KS and DirectSound.

All results are subject to variability depending on one's hardware, as illustrated in Table 3. Here we compare a variety of Windows computers and sound cards, under WDM/KS and DirectSound. The former shows much better hardware-invariance than the latter: the standard deviation across the five different onboard sound-cards' mean latencies is a mere 1.3 ms under WDM/KS, but 12.4 ms under DirectSound. The results also illustrate that a dedicated sound-card may give slight advantages (compare 3F vs. 3G), but this advantage may be undone if one chooses the wrong host API for the hardware's driver (the same comparison backfires under DirectSound: 3M vs. 3N). We can also see that some low-cost multi-channel USB devices can be used with little disadvantage (3D vs 3E, 3K vs. 3L). Our results from the same device under macOS and Linux, (not shown) also suggest that the USB latencies differ only slightly from onboard latencies.

**Table 3:**
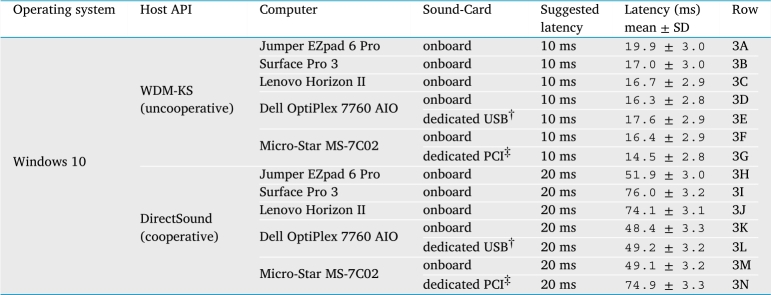
This table shows observed audio latency and jitter (mean and standard deviation of latency across 100 repetitions) measured via the headphone sockets of various Windows computers with various different sound cards, using the method described in Section 4. Two host APIs are compared: WDM/KS, our choice for low-latency applications, shows little variability between setups; by contrast DirectSound, our choice for cooperative applications, has much higher variability. For each host API, a suggested-latency value was chosen to be as low as possible, but high enough to avoid quality problems on *all* of the hardware setups. The sound-card marked ^†^ was a StarTech ICUSBAUDIO7D. The sound-card marked ^‡^ was a Creative Sound Blaster Audigy 5/Rx—before measuring it, we entered the computer's firmware settings and disabled the onboard sound-card so that the Sound Blaster was the only recognized sound output device.

Finally, Table 4 shows the results across three different operating systems. We note that, given the right choice of settings, it is possible to achieve low latency without artifacts on all of them (4C, 4F, 4N, 4S, 4X). On macOS, low latencies can even be achieved without sacrificing cooperativeness.^11^ One other observation arising from this table is that **PsychPortAudio** may lack hardware-invariance under Windows: recall that on our Dell computer, its best performance (2D) came reasonably close to the overall best (2B) but now we see that on the Surface Pro it did not (4J and 4K are nowhere near 4N, 4O and 4P). Under Linux and macOS, its performance is indistinguishable from plain **PortAudio**. However, given a sufficiently high target latency for the particular hardware, **PsychPortAudio**'s outstanding contribution lies in its pre-scheduling function, which achieves very low jitter on all platforms (4D, 4L, 4U).

**Table 4:**
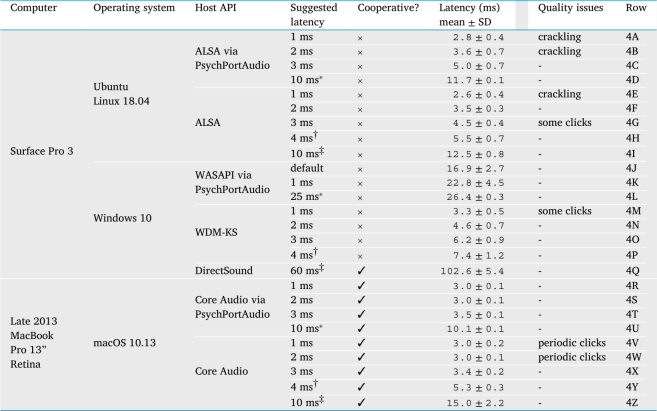
This table shows observed audio latency and jitter (mean and standard deviation of latency across 100 repetitions) measured via the headphone sockets of two portable computers running three different operating systems, using the method described in Section 4. In all configurations, it was possible to achieve low latencies (under 10 ms) without audible artifacts. “Suggested latency” refers to a configurable parameter exposed by the **PortAudio** library and by **audiomath** when initializing sound hardware, except where marked with ^⁎^: these entries were measured by adopting the best previous suggested-latency setting, but then using **PsychPortAudio**'s unique pre-scheduling functionality to configure a longer latency of 10 ms (or 25 ms for the Surface Pro under Windows, where **PsychPortAudio** could not achieve latencies under 10 ms). For each platform, the **LowLatencyMode(True)** default configuration is marked ^†^, and the more-robust **LowLatencyMode(False)** default configuration is marked ^‡^.

## 6 Discussion

We have documented the ways in which **audiomath** enables Python programmers to gather raw material to make auditory stimuli, to compose and tailor stimuli in memory, to present stimuli, and to record experimental sessions. These tasks entail manipulation, generation, visualization, decoding, encoding, recording and playback of audio data. It is possible to find many existing software tools—some programmable, some not—that offer one or more of these functions. The main advantage of **audiomath** is that it provides a single programmer's interface that unifies them all, with minimal third-party dependencies.

Among these tasks, the one that tends to consume the greatest amount of time and energy during development is stimulus presentation—especially the question of precise timing. The **audiomath** package provides new tools for addressing this challenge, and for measuring performance. We used these tools to measure audio latency and jitter across a variety of hardware, operating systems, and settings. The results show that, given the right choices, low latency (less than 10 ms) and low jitter (less than 1 ms) can be achieved even with non-specialized off-the-shelf computer hardware. Nonetheless these good results are part of a complex landscape containing many traps for the unwary. While retaining flexibility in case the programmer should require it, **audiomath** simplifies the problem by facilitating the choice of priorities between low latency, low jitter, or cooperativeness and robustness.

Note that the tests reported here were limited to consumer-grade sound processing cards—most of them “on-board”, i.e. integrated into the computer's main system board. It is encouraging to find that even these inexpensive cards can achieve low enough latencies for most neuroscientific intents and purposes. However, note that we have not yet been able to report any results representing the de-facto standard for low-latency audio, which is to use a professional-grade sound processor with a driver that supports the ASIO host API natively (i.e. not via ASIO4ALL) on Windows. So far, budget limitations have prevented us from testing hardware of high enough quality to assess the difference this might make. It would be valuable to extend our tests to more-expensive higher-quality sound processors, to assess the extent to which they might ease or remove some of the performance trade-off dilemmas.

For some of the core audio functions—specifically visualization, decoding, encoding, recording and playback—the bulk of the code that **audiomath** uses “under the hood” is not our own. The tools we wrote for these purposes, while often being non-trivial works in their own right, are really wrappers around functions from trusted third-party libraries. We have described the way in which these dependencies are managed in as conservative and modular a way as possible—for example, if the third-party visualization library **matplotlib** has or develops some incompatibility with your system, your **audiomath** applications that do *not* perform visualization (let's say they only need to perform playback) will still work. Such behavior cannot generally be assumed with scientific Python packages, which tend to sit on top of deep hierarchies of dependencies and be catastrophically vulnerable to weaknesses in any one of them.

By contrast, when it comes to generation and manipulation of audio content, **audiomath** provides original implementations. The central goal in their design was simplicity of use—hence, adding two sound objects' signals together is as simple as writing **x + y**, doubling a sound's amplitude is as simple as x *= 2, etc. The common operations of splicing (concatenating sounds together in time), and multiplexing (stacking channels to create multi-channel sounds) can also be performed with minimal typing, using the **%** and **&** operators, respectively.^12^ In implementing these operations, an important design goal was to minimize fuss. For example, when adding two sounds to superimpose them, or multiplying them to perform amplitude modulation, or multiplexing them together, **audiomath** does not complain about sounds being of unequal duration—it automatically pads shorter sounds to the required length. Similarly, when adding sounds, multiplying sounds, or splicing sounds along the time axis together, **audiomath** does not complain if one of the arguments is monophonic and the other has multiple channels—instead, it automatically replicates the monophonic content up to the required number of channels. This tolerant approach greatly reduces the complexity of the code that users have to write.

It is worth noting where the scope of **audiomath**'s functionality stops, relative to some other packages. We do not aim to perform sophisticated signal-processing in **audiomath**, preferring to leave the implementation details to more specialized packages such as **scipy.signal**, **librosa**, or dedicated speech-processing toolboxes. It also currently requires sound files to be completely loaded into memory before playback, lacking the functionality for streaming sounds from a file or network in the way offered by (for example) the **pyglet** package. This may be added in future versions.

## Declarations

### Author contribution statement

N. Jeremy Hill: Conceived and designed the experiments; Performed the experiments; Analyzed and interpreted the data; Contributed reagents, materials, analysis tools or data; Wrote the paper.

Scott W.J. Mooney: Performed the experiments; Contributed reagents, materials, analysis tools or data; Wrote the paper.

Glen T. Prusky: Contributed reagents, materials, analysis tools or data; Wrote the paper.

### Funding statement

This work was supported by institutional funding from Blythedale Children's Hospital, and by the National Center for Adaptive Neurotechnologies (10.13039/100000002NIH grant 7-P41-EB018783-07, National Institute for Biomedical Imaging and Bioengineering).

### Data availability statement

Data associated with this study has been deposited at https://osf.io/v6dwh/.

### Declaration of interests statement

The authors declare no conflict of interest.

### Additional information

No additional information is available for this paper.

## Footnotes

1 For background details regarding Windows audio APIs, see Kirk [13].

2 WASAPI stands for Windows Audio Session Application Programmer's Interface.

3 Python in particular may exacerbate the effects of resource contention between threads of the same process, because of its so-called Global Interpreter Lock [15].

4 ASIO stands for Audio Stream Input/Output.

5 WDM/KS stands for Windows Driver Model/Kernel Streaming.

6 MME stands for Multi-Media Extensions and is sometimes also known as WinMM.

7 The definition of “low enough” may differ in different applications—for example, ±5 ms may be acceptable when analyzing late or even mid-latency event-related potentials in EEG, but not when attempting to distinguish components of early brainstem evoked responses that are only one or two milliseconds apart.

8 Our shorthand for describing this effect is the “Pink Floyd problem”, referring to fact that many of that band's albums start with such a gradual fade-in that they cause a period of uncertainty as to whether or not one actually pressed “play”.

9 Luckily, it is generally safe to assume precise time-locking between the audible channels and the artificial sounds—precise inter-channel timing is a mission-critical feature of even the cheapest soundcards.

10 Naturally the user still has the option to override either set of defaults, as well as the choice of the third path of loading the **PsychPortAudio** back-end (which defaults to using the WASAPI host API on Windows) if low jitter is the overriding priority.

11 On Linux, the Advanced Linux Sound Architecture (ALSA) host API can also be used in a cooperative mode, in which we have found latency to be very high (ca. 150 ms) on our Surface Pro, but encouragingly lower on other hardware (ca. 8 ms on our Lenovo Horizon II).

12 When dealing with sound arrays, we have never come across meaningful applications of the modulo division operator (the conventional meaning of **%** in Python) or bitwise logical AND (the conventional meaning of **&**), so confusion is very unlikely in typical use-cases provided one is already familiar with **audiomath**'s conventions. However, in situations where familiarity cannot be assumed, use of such redefined operators is likely to reduce code readability. For this reason, functional forms **Concatenate()** and **Stack()** are also available, either as global functions (equivalent to **%** and **&** respectively) or as object methods (equivalent to **%=** and **&=**).
